# In Memoriam: James Donald Lafontaine 8 December 1948 — 20 November 2025

**DOI:** 10.3897/zookeys.1267.183799

**Published:** 2026-01-30

**Authors:** Christian Schmidt

**Affiliations:** 1 Agriculture and Agri-Food Canada, Ottawa, Canada Agriculture and Agri-Food Canada Ottawa Canada

Dr. J. Donald Lafontaine (Don to those who knew him) passed away on 20 November 2025, shortly after suffering from a stroke. Don was born at the Civic Hospital, Ottawa, Ontario, and grew up in Canada’s capital region. After graduating Nepean High School, Don pursued an undergraduate degree at Carleton University under Henry F. Howden (1972). From an early age, Don was passionate about nature, learning the local flora and fauna. He could find and identify plants, mammals, birds, reptiles, and amphibians. Nevertheless, it was his interest in butterflies and moths that would shape his career, despite briefly considering a career path in fern taxonomy.


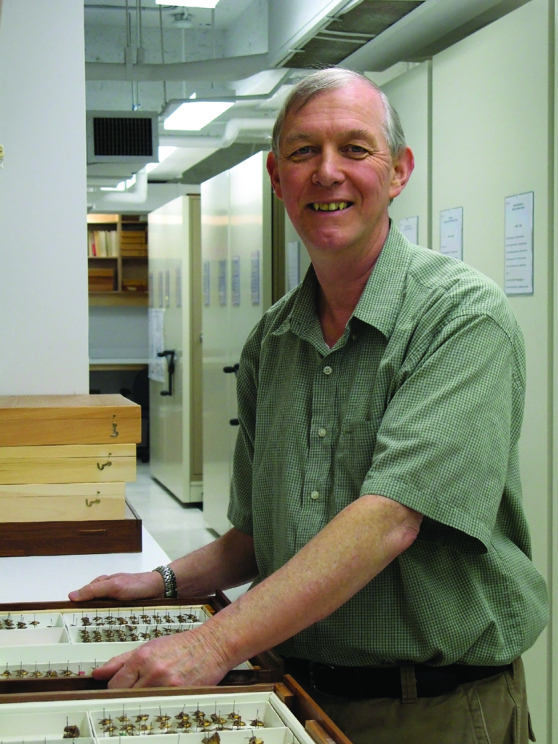


Don’s involvement in the local naturalist community and interest in Lepidoptera inevitably led to an introduction to the scientists at the Canadian National Collection of Insects, Arachnids and Nematodes (CNC), just a few miles from his home. Tom Freeman, Eugene Munroe and especially David F. Hardwick, then director of the Biosystematics Research Institute, would become Don’s mentors and career advocates. Influenced by David Hardwick and his work on cutworm and earworm moths, Don would enroll in a MSc program focusing on *Euxoa* cutworms under the guidance of George Ball at the University of Alberta. Nearing the end of his two-year program, George Ball advised that Don’s thesis essentially amounted to a PhD, and encouraged him to change programs, with an extra year to meet course requirements. Don completed his PhD in 1979, and shortly thereafter started his career as a research scientist at the CNC, following Hardwick’s retirement in 1978. Having already worked as a student at the CNC as early as 1971, Don’s “home base” at the CNC would span 46 years, during which time he became a leading world-authority on Owlet moths.

Don was author or co-author of over 130 scientific publications. Starting in 1968 at the age of 19, he published a checklist of Ottawa area butterflies, and two years later he described his first species new to science, a local hairstreak butterfly he named *Strymon borealis*. Subsequently synonymized under a more widespread species (*Satyrium calanus*), Don’s hairstreak literally flew off the taxonomy radar for the next 50 years. It was not until a week after Don’s death that a colleague pointed out that an overlooked hairstreak species seems to be present in eastern Canada, based on mtDNA barcode results. Incredibly, this hidden species was the very same taxon that Don described – a realization brought on solely by the fact that I had been compiling Don’s complete bibliography!

Don has left an exceptional scientific legacy in the study of noctuid moths. His mastery of North American cutworm moth taxonomy was unparalleled, particularly his early-career revisions of the genus *Euxoa*, arguably the most difficult and speciose group of Nearctic macrolepidopterans. Don would go on to author three monograph volumes, treating the entire North American Noctuini fauna in the “Moths of North America” series. Early on, Don also traveled extensively throughout western North America, and his first visit to the Yukon sparked a special interest in the Lepidoptera fauna of Beringia and tundra Lepidoptera. The mountain tundra of the Yukon was among Don’s favorite field experiences, and a rare Yukon tiger moth (still only known from the two type specimens) now bears his name, *Chelis lafontainei* (Ferguson, 1985) (Fig. 1). Later in his career, Don drew on his vast knowledge of Lepidoptera morphology to re-cast the higher classification of Noctuoidea, together with Michael Fibiger. This stands as a foundational framework for Noctuoidea classification today.

**Figure 1.**
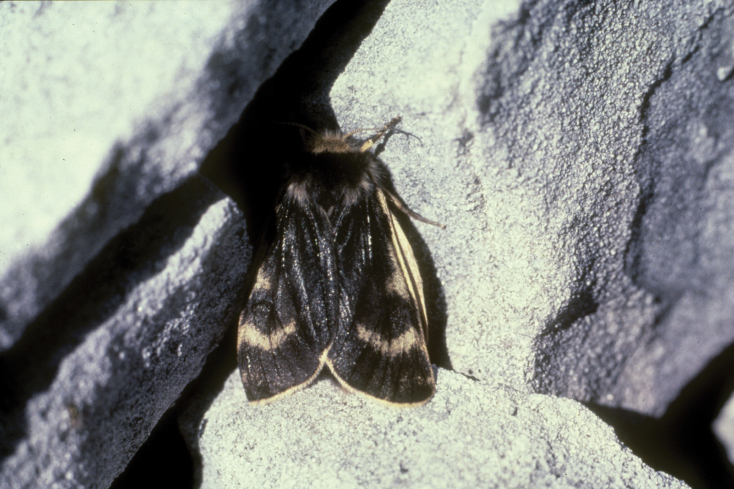
Allotype female of *Chelis lafontainei* at the type locality, Windy Pass, Yukon, Canada.

Naturally, Don received many accolades for his achievements in Lepidopterology, including invitations as symposium chair or keynote speaker in conferences that took him from Denver and Boston to Helsinki and Leningrad. In 2013, Don received the Queen Elizabeth II Diamond Jubilee Medal which honours those who have made a significant contribution to fellow Canadians, their community, or to Canada over the previous sixty years. He also received the Karl Jordan Medal in 2011, awarded for original research in Lepidoptera by The Lepidopterists’ Society. Don also served as editor for numerous journals, including The Canadian Entomologist, ZooKeys, and the “Moths of North America” series. In 2001, he served as president of The Lepidopterists’ Society, and he was a honorary life member of the Society for European Lepidopterology. Don valued the importance of nature conservation and also volunteered as a Lepidoptera expert with the Committee on the Status of Endangered Wildlife in Canada (COSEWIC). His vast biological knowledge and critical thinking skills greatly aided conservation efforts towards Canada’s insect fauna.

Over his long and prodigious career Don authored or co-authored more than 130 publications. He described 249 taxa, including 15 new genera and four family-group taxa. He is honoured by nine patronyms: eight species and one genus. One of the few remaining taxonomists trained and highly skilled in morphology, Don became an expert in genitalic morphology and dissection techniques; his legacy of dissection slides numbers over 7000.

Perhaps most significantly, Don was a gracious mentor to many people, always taking the time to teach and aid those who came to him for his vast experience. Recognizing the importance of those early mentors helping out an “amateur”, Don reciprocated on this philosophy, helping amateurs and professionals alike throughout his career. His generosity, expertise and knowledge will be greatly missed.

## Publications

Lafontaine JD (1968) The butterflies of the Ottawa region. Trail and Landscape 2(4): 94–97.

Lafontaine JD (1969) Take another look at hairstreaks. Trail and Landscape 3(5): 151.

Lafontaine JD (1970) A redescription of *Strymon borealis* Lafontaine (Lycaenidae). Journal of the Lepidopterists’ Society 24: 83–86.

Lafontaine JD, Brunton DF (1972) The Purple Cliff-brake, *Pellaea atropurpurea* (L.) Link, in western Quebec. Canadian Field-Naturalist 86: 297–298. https://doi.org/10.5962/p.343621

Lafontaine JD (1972) More butterflies. Trail and Landscape 6(3): 94–95.

Lafontaine JD (1973) Eastern North American species of *Antispila* (Lepidoptera: Heliozelidae) feeding on *Nyssa* and *Cornus*. The Canadian Entomologist 105(7): 991–996. https://doi.org/10.4039/Ent105991-7

Brunton DF, Lafontaine JD (1974) An unusual escarpment flora in western Quebec. Canadian Field Naturalist 88: 337–344. https://doi.org/10.5962/p.344416

Brunton DF, Lafontaine JD (1974) The distribution of *Pellaea* in Quebec and eastern Ontario. Naturaliste Canadien 101: 937–939.

Lafontaine JD (1974) The *punctigera* group ofthe genus *Euxoa* (Lepidoptera: Noctuidae) with descriptions of two new species. The Canadian Entomologist 106: 1233–1240. https://doi.org/10.4039/Ent1061233-12

Lafontaine JD (1974) A new species of *Coptodisca* (Heliozelidae) from Mississippi on Farkleberry (*Vaccinium arboreum*). Journal of the Lepidopterists’ Society 28: 126–130.

Lafontaine JD (1974) A new species of *Euxoa* (Lepidoptera: Noctuidae) from western United States. The Canadian Entomologist 106(6): 651–654. https://doi.org/10.4039/Ent106651-6

Lafontaine JD (1974) A synopsis of the redimicula group of the genus *Euxoa* Hbn. (Lepidoptera: Noctuidae) with a computer analysis of genitalic characters. The Canadian Entomologist 106(4): 409–421. https://doi.org/10.4039/Ent106409-4

Lafontaine JD (1975) The *mimallonis* group of the genus *Euxoa* Hbn. (Lepidoptera: Noctuidae) with descriptions of three new species. The Canadian Entomologist 107: 155–165. https://doi.org/10.4039/Ent107155-2

Lafontaine JD (1975) The *misturata* group of the genus *Euxoa* (Lepidoptera: Noctuidae) with a description of a new species. The Canadian Entomologist 107(12): 1327–1332. https://doi.org/10.4039/Ent1071327-12

Byers JR, Hinks CF, and Lafontaine JD (1975) Biosystematics of the genus *Euxoa* (Lepidoptera: Noctuidae) II. A description of the immature stages of *Euxoa basalis* and a redescription of the adult. The Canadian Entomologist 107: 1083–1094. https://doi.org/10.4039/Ent1071083-10

Lafontaine JD (1976) Two new species of *Euxoa* (Lepidoptera: Noctuidae) from southern United States allied to *Euxoa camalpa*. The Canadian Entomologist 108: 663–668. https://doi.org/10.4039/Ent108663-6

Lafontaine JD (1976) A synopsis of the *aequalis* group of the genus *Euxoa* Hbn. (Lepidoptera: Noctuidae) with a description of a new species from southwestern United States. The Canadian Entomologist 108: 741–750. https://doi.org/10.4039/Ent108741-7

Lafontaine JD (1976) A new species of *Euxoa* (Lepidoptera: Noctuidae) allied to *Euxoa olivia* and *Euxoa septentrionalis*. The Canadian Entomologist 108: 1275–1280. https://doi.org/10.4039/Ent1081275-11

Rockburne EW, Lafontaine JD (1976) The cutworms of Ontario and Quebec. Canada Department of Agriculture, Research Branch, Publication 1593, 164 pp.

Cook FR, Lafontaine JD, Black S, Luciuk L, Lindsay RV (1980) Spotted turtles (*Clemmys guttata*) in eastern Ontario and adjacent Quebec. Canadian Field-Naturalist 94(4): 411–415. https://doi.org/10.5962/p.347130

Kendall DM, Kevan PG, Lafontaine JD (1981) Nocturnal flight activity of moths (Lepidoptera) in alpine tundra. The Canadian Entomologist 113(7): 607–614. https://doi.org/10.4039/Ent113607-7

Lafontaine JD (1981) Classification and phylogeny of the *Euxoa detersa* group (Lepidoptera: Noctuidae). Quaestiones Entomologicae 17: 1–120.

Layberry RA, Lafontaine JD, Hall PW (1981) Butterflies: some old, some new, some help from you. Trail and Landscape 15(3): 118–122.

Byers JR, Lafontaine JD (1982) Biosystematics of the genus *Euxoa* (Lepidoptera: Noctuidae) XVI. Comparative biology and experimental taxonomy of four subspecies of *Euxoa comosa*. The Canadian Entomologist 114: 551–565. https://doi.org/10.4039/Ent114551-7

Lafontaine JD (1982) *Agrotis redimicula* Morrison, 1875 (Insecta: Lepidoptera): proposed conservation from 1874. Z.N.(S.) 2305. The Bulletin of Zoological Nomenclature 39: 54–56.

Lafontaine JD (1982) Biogeography of the genus *Euxoa* (Lepidoptera: Noctuidae) in North America. The Canadian Entomologist 114: 1–53. https://doi.org/10.4039/Ent1141-1

Lafontaine JD, Franclemont JG, Ferguson DC (1982) Classification and life history of *Acsala anomala* (Arctiidae: Lithosiinae). Journal of the Lepidopterists’ Society 36: 218–226.

Lafontaine JD, Byers JR (1982) A revision of the comosa group of the genus *Euxoa* Hbn. (Lepidoptera: Noctuidae) with descriptions of two new species. The Canadian Entomologist 114: 575–589. https://doi.org/10.4039/Ent114575-7

Kononenko VS, Lafontaine JD, Mikkola K (1983) A revision of the genus *Xestia* subg. *Schoyenia* Auriv. (Lepidoptera: Noctuidae), with descriptions of four new species and a new subspecies. Insect Systematics & Evolution 14(3): 337–369. https://doi.org/10.1163/187631283X00353

Layberry RA, Lafontaine JD, Hall PW (1982) Butterflies of the Ottawa district. Trail and Landscape 16(1): 3–56.

Layberry RA, Lafontaine JD, Hall PW (1983) Butterflies of the Ottawa district, 1982 update. Trail and Landscape 17(3): 133–138.

Tshistjakov YA, JD Lafontaine (1984) A review of the genus *Dodia* Dyar (Lepidoptera: Arctiidae) with description of a new species from eastern Siberia and northern Canada. The Canadian Entomologist 116: 1549–1556. https://doi.org/10.4039/Ent1161549-11

Byers JR, Struble DL, and Lafontaine JD (1985) Biosystematics of the genus *Euxoa* (Lepidoptera: Noctuidae) XVIII. Comparative biology and experimental taxonomy of the sibling species *Euxoa ridingsiana* (Grt.) and *Euxoa maimes* (Sm.). The Canadian Entomologist 117: 481–493. https://doi.org/10.4039/Ent117481-4

Catling PM, Lafontaine JD (1986) First documented record of *Oarisma powesheik* (Lepidoptera: Hesperiidae) in Canada. The Great Lakes Entomologist 19(1): 2. https://doi.org/10.22543/0090-0222.1557

Lafontaine JD (1986) Identity of “*Autographa*” *ottolenguii* Dyar and occurrence of *Autographa buraetica* (Staudinger) in North America (Noctuidae: Plusiinae). Journal of the Lepidopterists’ Society 40: 158–163.

Lafontaine JD, Kononenko VS (1986) A revision of the genus *Trichosilia* Hampson (Lepidoptera: Noctuidae) with descriptions of four new species. The Canadian Entomologist 118: 1079–1113. https://doi.org/10.4039/Ent1181079-11

Mikkola K, Lafontaine JD (1986) A preliminary note on the taxonomy of the *Apamea zeta* complex, with a first report of *A. zeta* from Fennoscandia (Lepidoptera, Noctuidae). Notulae Entomologicae 66: 91–95.

Lafontaine JD, Kononenko VS, McCabe TL (1986) A review of the *Lasionycla leucocycla* complex (Lepidoptera: Noctuidae) with descriptions of three new subspecies. The Canadian Entomologist 118: 255–279. https://doi.org/10.4039/Ent118255-3

Lafontaine JD (1987) The Moths of America north of Mexico. Noctuoidea, Noctuidae (part) – *Euxoa*. Fascicle 27.2. The Wedge Entomological Research Foundation, Washington, 237 pp.

Lafontaine JD, Mikkola K, Kononenko VS (1987) A revision of the genus *Xestia* subg. *Pachnobia* (Lepidoptera: Noctuidae), with descriptions of two new subspecies. Entomologica Scandinavica 18: 305–331.

Lafontaine JD (1987) Noctuoidea: Noctuidae: Noctuinae (Part) – *Euxoa*. Fascicle 27.2. In: RB Dominick, DC Ferguson, JG Franclemont, RW Hodges, EG Munroe (eds) The Moths of America North of Mexico. Wedge Entomological Research Foundation, Washington, DC, 237 pp. https://images.peabody.yale.edu/mona/27-2-ocr.pdf

Lafontaine JD Mikkola K (1987) Lock-and-key systems in the inner genitalia of Noctuidae (Lepidoptera) as a taxonomic character. Entomologiske Meddelelser 55: 161–167.

Lafontaine JD, Mikkola K, Kononenko VS (1987) *Anarta cordigera* (Thunberg) (Lepidoptera: Noctuidae), a species complex. The Canadian Entomologist 119: 931–940. https://doi.org/10.4039/Ent119931-10

Lafontaine JD, Allyson S, Behan-Pelletier VM, Borkent A, Campbell JM, Hamilton KGA, Martin JEH, Masner L (Eds.) (1987) The insects, spiders and mites of Cape Breton Highlands National Park. BRC Report 1, Agriculture Canada, Ottawa, 302 pp.

Mikkola KE, Lafontaine JD, Grotenfelt P (1987) A Holarctic revision of the *Chersotis andereggii* complex (Lepidoptera: Noctuidae). Nota Lepidopterologia 10: 140–157.

Lafontaine JD, Kononenko VS (1988) A review of the genus *Parabarrovia* Gibson (Lepidoptera: Noctuidae) with description of the immature stages and a new species. The Canadian Entomologist 120: 507–523. https://doi.org/10.4039/Ent120507-6

Lafontaine JD, Kononenko VS (1988) A revision of the *Lasionycta skraelingia* (Herrich-Schäffer) species complex (Lepidoptera: Noctuidae). The Canadian Entomologist 120: 903–916. https://doi.org/10.4039/Ent120903-10

Kononenko VS, Lafontaine JD, Mikkola K (1989) An annotated check list of noctuid moths (Lepidoptera, Noctuidae) of Beringia. Entomologicheskoe Obozrenie 68: 549–567. Reprinted in English in Entomological Review 69: 17–138.

Ahola M, Lafontaine JD (1990) Larvae of *Xestia kolymae* (Herz) and *X. lorezi* (Staudinger)(Lepidoptera: Noctuidae), with notes on the geographical variation of the latter. Insect Systematics & Evolution 21(1): 77–90. https://doi.org/10.1163/187631290X00067

Byers JR, Struble DL, Herle CE, Kozub GC, Lafontaine JD (1990) Electroantennographic responses differentiate sibling species of dingy cutworm complex, *Feltia jaculifera* (Gn.) (Lepidoptera: Noctuidae). Journal of Chemical Ecology 16(10): 2969–2980. https://doi.org/10.1007/BF00979488

Koponen S, Lafontaine JD (1991) Noctuidae (Lepidoptera) from Kuujjuarapik, Northern Québec. Naturaliste Canadien 118(1): 63–65. https://www.provancher.org/wp-content/uploads/2025/03/Naturaliste_Canadien_V118_1991_compressed.pdf#page=65

Lafontaine JD, Poole RW (1991) *Plusia flacifera* Kirby, 1837 (currently *Anagrapha falcifera*; Insecta, Lepidoptera): proposed conservation of the specific name. Bulletin of Zoological Nomenclature 48: 41–42. https://doi.org/10.5962/bhl.part.670

Lafontaine JD, Poole RW (1991) Noctuoidea: Noctuidae: Plusiinae. Fascicle 25.1. In: R.B. Dominick, D.C. Ferguson, J.G. Franclemont, R.W. Hodges, & E.G. Munroe (eds). The Moths of America North of Mexico. Wedge Entomological Research Foundation, Washington, DC, 182 pp. https://images.peabody.yale.edu/mona/25-1-ocr.pdf

Mikkola K, Lafontaine JD, Kononenko VS (1991) Zoogeography of Holarctic species of Noctuidae (Lepidoptera): importance of the Beringian Refuge. Entomologica Fennica 2: 1–17. https://doi.org/10.33338/ef.83545

Troubridge JT, Fitzpatrick SM, Lafontaine JD (1992) *Apamea ophiogramma* (Esper), a Palearctic cutworm new to North America (Lepidoptera: Noctuidae). The Canadian Entomologist 124: 109–112. https://doi.org/10.4039/Ent124109-1

Lafontaine JD (1993) Cutworm systematics: confusions and solutions. The Memoirs of the Entomological Society of Canada 125(S165): 189–196. https://doi.org/10.4039/entm125165189-1

Mikkola K, Fibiger M, Lafontaine JD (1994) Revision of the *Xestia speciosa* and *X. alpicola* complexes in Europe (Lepidoptera, Noctuidae). Entomologica Fennica 5(2): 125–128. https://doi.org/10.33338/ef.83804

Mikkola K, Lafontaine JD (1994) Recent introductions of riparian noctuid moths from the Palearctic Region to North America, with the first report of *Apamea unanimis* (Hübner) (Noctuidae: Amphipyrinae). Journal of the Lepidopterists’ Society 48: 121–127.

Crolla JP, Lafontaine JD (1996). COSEWIC Status Report on the Monarch Butterfly, 23 pp.

Kononenko VS, Lafontaine JD, Mikkola K (1996) Taxonomy and zoogeography of some arctic Noctuidae (Lepidoptera), with descriptions of three new species and one new subspecies. Acta Zoolologica Fennica 200: 83–94.

Lafontaine JD (1996) Butterflies and moths (Lepidoptera). In: I.M. Smith (ed.) Species diversity in the Mixedwood Plains Ecozone. Ecological Monitoring and Assessment Network. Burlington, ON, 28 pp.

Crabo LG, Lafontaine JD (1997) A revision of the *cornuta* group of *Cerastis* subgenus *Metalepsis* (Noctuidae). Journal of the Lepidopterists’ Society 51: 237–248. https://images.peabody.yale.edu/lepsoc/jls/1990s/1997/1997-51(3)237-Crabo.pdf

Handfield L, Landry J-F, Landry B, Lafontaine JD (1997) Liste des Lépidoptères du Quebec et du Labrador [List of the Lepidoptera of Quebec and of Labrador]. Fabreries (Supplement) 7: 1–155.

Lafontaine JD, Wood DM (1997) Butterflies and moths (Lepidoptera) of the Yukon. In: HV Danks & JA Downes (eds) Insects of the Yukon. Biological Survey of Canada (Terrestrial Arthropods), Ottawa, ON, 723–785 https://www.ualberta.ca/en/biological-sciences/media-library/services/strickland/yukon.pdf

Layberry RA, Hall PW, Lafontaine JD (1998) The Butterflies of Canada. NRC Research Press, Canada Institute for Scientific and Technical Information, in association with University of Toronto Press, Toronto, ON, 280 pp. https://doi.org/10.3138/9781442623163

Lafontaine JD (1998) Noctuoidea: Noctuidae (part): Noctuinae: Noctuini. Fascicle 27.3. In: RB Dominick, DC Ferguson, JG Franclemont, RW Hodges, EG Munroe (eds) The Moths of America North of Mexico. Wedge Entomological Research Foundation, Washington, DC, 348 pp. https://images.peabody.yale.edu/mona/27-3-ocr.pdf

Fitzpatrick SM, Troubridge JT, Henderson D (2000) *Ochropleura implecta* (Lepidoptera: Noctuidae), a new cutworm pest of cranberries. The Canadian Entomologist 132(3): 365–367. https://doi.org/10.4039/Ent132365-3

Mustelin T, Leuschner R, Mikkola K, Lafontaine JD (2000) Two new genera and thirteen new species of owlet moths (Lepidoptera: Noctuidae), mainly from Southern California. Proceedings of the San Diego Society of Natural History 36: 1–18. https://brccapi.sdnhm.org/files/6613/6520/6710/Proceedings36_2000_Mustelin_Leuschner_Mikkola__Lanfontaine.pdf

Lafontaine JD, Dickel TS (2000) A New *Autographa* from Colorado (Lepidoptera: Noctuidae: Plusiinae). Holarctic Lepidoptera 7(2): 49–50. https://journals.flvc.org/holarctic/article/download/90477/86777

Lafontaine JD, Troubridge JT (2000) Two new species of Arctiidae (Lepidoptera) from the Yukon Territory, Canada. Journal of the Entomological Society of British Columbia 96: 89–93. https://journal.entsocbc.ca/index.php/journal/article/view/492/502

Lafontaine, JD (2001) Presidential profile: Don Lafontaine. News of the Lepidopterists’ Society 43(4): 102.

Lafontaine JD, Troubridge JT, Thomas AW (2001) Moths and butterflies (Lepidoptera) of the Atlantic Maritime Ecozone. In: D.F. McAlpine & I.M. Smith (eds) Assessment of species diversity in the Atlantic Maritime Ecozone. NRC Research Press, Ottawa, ON, 489–537.

Troubridge JT, Lafontaine JD (2002) Revision of the species of the “*Oligia*” *semicana* group (Lepidoptera: Noctuidae) with descriptions of a new genus and 12 new species. The Canadian Entomologist 134: 157–191. https://doi.org/10.4039/Ent134157-2

Lafontaine JD, Mikkola K (2003) New species of *Xanthia* (Lepidoptera: Noctuidae) from North America. The Canadian Entomologist 135: 549–554. https://doi.org/10.4039/n02-116

Lafontaine JD, Troubridge JT (2003) Review of the genus *Cosmia* (Lepidoptera: Noctuidae) in North America, with description of a new species. The Canadian Entomologist 135: 325–336. https://doi.org/10.4039/n02-055

Troubridge JT, Lafontaine JD (2003) A review of the pine-feeding *Lithophane lepida* species group (Lepidoptera: Noctuidae), with descriptions of two new species. The Canadian Entomologist 135: 53–62. https://doi.org/10.4039/n02-041

Lafontaine JD (2004) Noctuoidea: Noctuidae (part): Noctuinae (part – Agrotini). Fascicle 27.1. In: R.W. Hodges, D.R. Davis, D.C. Ferguson, E.G. Munroe, and J.A. Powell (editors). The Moths of America North of Mexico. Wedge Entomological Research Foundation, Washington, DC, 385 pp. https://images.peabody.yale.edu/mona/27-1-ocr.pdf

Lafontaine, J.D. & Troubridge, J.T. 2004. Description of a new genus and two new species of cutworm moths (Lepidoptera: Noctuidae). The Canadian Entomologist 136: 823–834. https://doi.org/10.4039/n04-038

Troubridge JT, Lafontaine JD (2004) Revision of the genus *Hyppa* (Lepidoptera: Noctuidae) with description of a new species. The Canadian Entomologist 136: 299–311. https://doi.org/10.4039/n03-070

Lafontaine JD, Fibiger M (2006) Revised higher classification of the Noctuoidea (Lepidoptera). The Canadian Entomologist 138: 610–635. https://doi.org/10.4039/n06-012

Troubridge JT, Lafontaine JD (2007) A revision of the North American species of *Brachylomia* (Lepidoptera: Noctuidae: Xyleninae) with descriptions of four new species. The Canadian Entomologist 139: 209–227. https://doi.org/10.4039/n06-044

Lafontaine JD, Dickel TS (2008) Review of the genus *Epidromia* in North America (Lepidoptera: Noctuidae: Catocalinae). Lepidoptera Novae 1: 109–115.

Lafontaine JD, Dickel TS, Schweitzer DF, McCabe TL, Metlevski J (2008) Taxonomy and identification of *Phoberia* species (Lepidoptera: Noctuidae: Catocalinae). Lepidoptera Novae 1: 103–108.

Adams JK, Lafontaine JD (2009) A new species of *Plagiomimicus* Grote (Lepidoptera: Noctuidae: Stiriinae). Journal of the Lepidoptera Society 63: 173–176. https://images.peabody.yale.edu/lepsoc/jls/2000s/2009/2009-63-3-173.pdf

Brou Jr VA., Lafontaine JD (2009) A new species of *Lithophane* Hbn. (Lepidoptera, Noctuidae, Xyleninae) from southeastern United States. ZooKeys 9: 11–20. https://doi.org/10.3897/zookeys.9.158

Crabo LG, Lafontaine JD (2009) A revision of *Lasionycta* Aurivillius (Lepidoptera, Noctuidae) for North America and notes on Eurasian species, with descriptions of 17 new species, 6 new subspecies, a new genus, and two new species of *Tricholita* Grote. ZooKeys 30: 1–155. https://doi.org/10.3897/zookeys.30.308

Ferris CD, Lafontaine JD (2009) Review of the *Acontia areli* group with descriptions of three new species (Lepidoptera, Noctuidae, Acontiinae). ZooKeys 9: 27–46. https://doi.org/10.3897/zookeys.9.180

Janzen DH, Hallwachs W, Blandin P, Burns JM, Cadiou JM, Chacon I, Dapkey T, Deans AR, Epstein ME, Espinoza B, Franclemont JG, Haber WA, Hajibabaei M, Hall JPW, Hebert PDN, Gauld ID, Harvey DJ, Hausmann A, Kitching IJ, Lafontaine D, Landry JF, Lemaire C, Miller JY, Miller JS, Miller L, Miller SE, Montero J, Munroe E, Green SR, Ratnasingham S, Rawlins JE, Robbins RK, Rodriguez JJ, Rougerie R, Sharkey MJ, Smith MA, Solis MA, Sullivan JB, Thiaucourt P, Wahl DB, Weller SJ, Whitfield JB, Willmott KR, Wood DM, Woodley NE, Wilson JJ (2009) Integration of DNA barcoding into an ongoing inventory of complex tropical biodiversity. Molecular Ecology Resources 8(Suppl 1): 1–26. https://doi.org/10.1111/j.1755-0998.2009.02628.x

Lafontaine JD, Dickel TS, Honey MR (2009) Taxonomy and identification of *Magusa* species (Lepidoptera: Noctuidae: Xyleninae). Lepidoptera Novae 2: 35–40.

Lafontaine JD, Honey MR (2009) Taxonomic changes to the names *Zanclognatha jaccusalis* and *Z. ochreipennis* (Lepidoptera: Noctuidae: Herminiinae). Lepidoptera Novae 2: 41–43.

Lafontaine JD, Sullivan JB (2009) A review of the genus *Megalographa* Lafontaine and Poole (Lepidoptera: Noctuidae: Plusiinae) with the description of a new species from Costa Rica. Insecta Mundi 0077: 1–10. https://digitalcommons.unl.edu/insectamundi/605/

Mikkola K, Lafontaine JD, Gill J (2009) Noctuoidea: Noctuidae (part): Noctuinae (part) Xyleninae (part) Apameini (part – *Apamea* group of genera). Fascicle 26.9. In: RW Hodges, RL Brown, DR Davis, JD Lafontaine, JA Powell, MA Solis (eds) The Moths of America North of Mexico. Wedge Entomological Research Foundation, Washington, DC, 192 pp. https://images.peabody.yale.edu/mona/26-9-ocr.pdf

Schmidt BC, Lafontaine JD (Editors). 2009. Contributions to the systematics of New World macro-moths. ZooKeys 9: 1–134. https://doi.org/10.3897/zookeys.9.183

Ferris CD, Lafontaine JD (2010) Review of the North American species of *Marimatha* Walker with descriptions of three new species (Lepidoptera, Noctuidae, Eustrotiinae) and the description of *Pseudomarimatha flava* (Noctuidae, Noctuinae, Elaphriini), a new genus and species confused with *Marimatha*. ZooKeys 39: 117–135. https://doi.org/10.3897/zookeys.39.424

Lafontaine JD, Troubridge JT (2010) Two new species of the *Euxoa westermanni* species-group from Canada (Lepidoptera, Noctuidae, Noctuinae). ZooKeys 39: 255–262. https://doi.org/10.3897/zookeys.39.436

Lafontaine JD, Troubridge JT, Thomas AW (2010) Moths and butterflies (Lepidoptera) of the Atlantic Maritime Ecozone. In: McAlpine DF, Smith IM (eds) Assessment of species diversity in the Atlantic Maritime Ecozone. NRC Research Press, Ottawa, ON, 489–537.

Lafontaine JD, Walsh JB, Holland RW (2010) A revision of the genus *Bryolymnia* Hampson in North America with descriptions of three new species (Lepidoptera, Noctuidae, Noctuinae, Elaphriini). ZooKeys 39: 187–204. https://doi.org/10.3897/zookeys.39.437

Lafontaine JD Walsh JB (2010) A review of the subfamily Anobinae with the description of a new species of *Baniana* Walker from North and Central America (Lepidoptera, Erebidae, Anobinae). ZooKeys 39: 3–11. https://doi.org/10.3897/zookeys.39.428

Lafontaine JD Ferris CDW, Walsh JB (2010) A revision of the genus *Hypotrix* Guenée in North America with descriptions of four new species and a new genus (Lepidoptera, Noctuidae, Noctuinae, Eriopygini). ZooKeys 39: 225–253. https://doi.org/10.3897/zookeys.39.438

Lafontaine JD Poole RW (2010) Review of the New World genera of the subfamily Acontiinae (Lepidoptera, Noctuidae). ZooKeys 39: 137–160. https://doi.org/10.3897/zookeys.39.427

Lafontaine JD Schmidt BC (2010) Annotated check list of the Noctuoidea (Insecta, Lepidoptera) of North America north of Mexico. ZooKeys 40: 1–239. https://doi.org/10.3897/zookeys.40.414

Schmidt BC, Lafontaine JD (Editors) (2010) Contributions to the systematics of New World macro-moths II. ZooKeys 39: 1–272. https://doi.org/10.3897/zookeys.39.442

Hall P W, Catling PM, Lafontaine JD (2011) Insects at risk in the prairie region. Arthropods of Canadian Grasslands 2: 323–349.

Lafontaine JD, Troubridge JT (2011) Moths and butterflies of the Montane Cordillera Ecozone. In: G.G.E. Scudder & I.M. Smith (editors). Assessment of species diversity in the Montane Cordillera Ecozone. Royal British Columbia Museum, Victoria, BC, 89 pp.

van Nieukerken E, Kaila L, Kitching I, Kristensen NP, Lees D, Minet J, Mitter J, Mutanen M, Regier J, Simonsen T, et al. (2011) Order Lepidoptera Linnaeus, 1758. Zootaxa 3148:212–221. https://doi.org/10.11646/zootaxa.3148.1.41

Lafontaine JD Schmidt BC (2011) Additions and corrections to the check list of the Noctuoidea (Insecta, Lepidoptera) of North America north of Mexico. ZooKeys 149: 145–161. https://doi.org/10.3897/zookeys.149.1805

Schmidt BC, Lafontaine JD (Editors). (2011) Contributions to the systematics of New World macro-moths III. ZooKeys 149: 1–161. https://doi.org/10.3897/zookeys.149.2383

Sullivan JB, Lafontaine JD (2011) New synonymies and combinations in *Argyrostrotis* Hübner (Lepidoptera, Erebidae, Erebinae, Poaphilini). ZooKeys 149: 107–116. https://doi.org/10.3897/zookeys.149.2347

Zahiri R, Kitching IJ, Lafontaine JD, Mutanen M, Kaila L, Holloway JD, Wahlberg N (2011) A new molecular phylogeny offers hope for a stable family-level classification of the Noctuoidea (Insecta: Lepidoptera). Zoologica Scripta 40: 158–173. https://doi.org/10.1111/j.1463-6409.2010.00459.x

Ferris CD, Kruse JJ, Lafontaine JD, Philip KW, Schmidt BC, Sikes DS (2012) A checklist of the moths of Alaska. Zootaxa 3571: 1–25. https://doi.org/10.11646/zootaxa.3571.1.1

Zahiri R, Holloway JD, Kitching IJ, Lafontaine JD, Mutanen M, Wahlberg N (2012) Molecular phylogenetics of Erebidae (Lepidoptera, noctuoidea). Systematic Entomology 37(1): 102–124. https://doi.org/10.1111/j.1365-3113.2011.00607.x

Lafontaine D, Schmidt C (2013) Comments on differences in classification of the superfamily Noctuoidea (Insecta, Lepidoptera) between Eurasia and North America. ZooKeys 264: 209–217. https://doi.org/10.3897/zookeys.264.4441

Lafontaine JD Schmidt BC (2013) Additions and corrections to the check list of the Noctuoidea (Insecta, Lepidoptera) of North America north of Mexico. ZooKeys 264: 227–236. https://doi.org/10.3897/zookeys.264.4443

Lafontaine JD, Walsh JB (2013) A revision of the genus *Ufeus* Grote with the description of a new species from Arizona (Lepidoptera, Noctuidae, Noctuinae, Xylenini, Ufeina). ZooKeys 264: 193–207. https://doi.org/10.3897/zookeys.264.3526

Schmidt BC, Lafontaine JD (2013) Lepidoptera family-group names proposed by Thaddeus William Harris in 1841. ZooKeys 264: 219–226. https://doi.org/10.3897/zookeys.264.4442

Schmidt BC, Lafontaine JD (Eds) (2013) Contributions to the systematics of New World macro-moths IV. ZooKeys 264: 1–238. https://doi.org/10.3897/zookeys.264.4687

Zahiri R, Lafontaine JD, Schmidt BC, Holloway JD, Kitching IJ, Mutanen M, Wahlberg N (2013) Relationships among the basal lineages of Noctuidae (Lepidoptera, Noctuoidea) based on eight gene regions. Zoologica Scripta 42: 488–507. https://doi.org/10.1111/zsc.12022

Zahiri R, Lafontaine JD, Holloway JD, Kitching IJ, Schmidt BC, Kaila L, Wahlberg N (2013) Major lineages of Nolidae (Lepidoptera, Noctuoidea) elucidated by molecular phylogenetics. Cladistics 1: 1–23. https://doi.org/10.1111/cla.12001

Metzler EH, Knudson EC, Poole RW, Lafontaine JD, Pogue MG (2013) A review of the genus *Ogdoconta* Butler (Lepidoptera, Noctuidae, Condicinae, Condicini) from North America north of Mexico with descriptions of three new species. ZooKeys 264: 165–191. https://doi.org/10.3897/zookeys.264.4060

Lafontaine JD, Walsh JB, Ferris CD (2014) A revision of the genus *Protorthodes* McDunnough with descriptions of a new genus and four new species (Lepidoptera, Noctuidae, Noctuinae, Eriopygini). ZooKeys 421: 139–179. https://doi.org/10.3897/zookeys.421.6664

Pohl GR, Schmidt BC, Lafontaine JD, Landry J-F, Anweiler GG, Bird CD (2014) Moths and butterflies of the Prairies Ecozone in Canada. In: DJ Giberson, HA Cárcamo (eds) Arthropods of Canadian grasslands (Volume 4): biodiversity and systematics Part 2. Biological Survey of Canada, Ottawa, ON, 169–238. http://biologicalsurvey.ca/monographs/read/17

Schmidt BC, Lafontaine JD (Editors) (2014) Contributions to the systematics of New World macro-moths V. ZooKeys 421: 1–191. https://doi.org/10.3897/zookeys.421.8050

Zahiri R, Lafontaine JD, Schmidt BC, DeWaard JR, Zakharov EV, Hebert PDN (2014) A transcontinental challenge – a test of DNA barcode performance for 1541 species of Canadian Noctuoidea (Lepidoptera). PLoS ONE 9: (3) e92797. https://doi.org/10.1371/journal.pone.0092797

Lafontaine JD, Schmidt BC (2015) Additions and corrections to the check list of the Noctuoidea (Insecta, Lepidoptera) of North America north of Mexico III. ZooKeys 527: 127–147. ttps://doi.org/10.3897/zookeys.527.6151

Lafontaine JD Sullivan JB (2015) A revision of the genus *Doryodes* Guenée, 1857, with descriptions of six new species (Lepidoptera, Erebidae, Catocalinae, Euclidiini). ZooKeys 527: 3–30. https://doi.org/10.3897/zookeys.527.6087

Schmidt BC, Lafontaine JD (Editors) (2015) Contributions to the systematics of New World macro-moths VI. ZooKeys 527: 1–147. https://doi.org/10.3897/zookeys.527.9686

Zahiri R, Lafontaine JD, Schmidt BC, deWaard JR, Zakharov EV, Hebert PDN (2017) Probing planetary biodiversity with DNA barcodes: the Noctuoidea of North America. PLOS ONE 12(6): e0178548. https://doi.org/10.1371/journal.pone.0178548

Miller JS, Wagner DL, Opler PA, Lafontaine JD (2018) Drepanoidea, Doidae, and Noctuoidea, Notodontidae (Part): Pygaerinae, Notodontinae, Cerurinae, Phalerinae, Periergosinae, Dudusinae, Hemiceratinae. Fascicle 22.1A. In: Lafontaine, JD (ed). The Moths of America North of Mexico. The Wedge Entomological Research Foundation, Washington, DC, 348 pp. https://images.peabody.yale.edu/mona/22-1A-ocr.pdf

Miller JS, Wagner DL, Opler PA, Lafontaine JD (2021) Noctuoidea, Notodontidae (Conclusion): Heterocampinae, Nystaleinae, Dioptinae, Dicranurinae. Fascicle 22.1A. In: Lafontaine, JD (ed). The Moths of America North of Mexico. The Wedge Entomological Research Foundation, Washington, DC, 443 pp. https://images.peabody.yale.edu/mona/22-1B-ocr.pdf

Pohl GR, Landry JF, Schmidt BC, Lafontaine JD, Troubridge JT, Macaulay AD, van Nieukerken EJ, deWaard JR, Dombroskie JJ, Klymko J, Nazari V, Stead K (2018) Annotated checklist of the moths and butterflies (Lepidoptera) of Canada and Alaska. Pensoft Series Faunistica 118: 1–580. https://repository.naturalis.nl/pub/648850/Pohl_et_al_2018_Checklist_Lepidoptera_Canada_Alaska.pdf

Schmidt BC, Lafontaine JD (Editors) (2018) Contributions to the systematics of New World macro-moths VII. ZooKeys 788: 1–252. https://doi.org/10.3897/zookeys.788.30148

Schmidt BC, Lafontaine JD, Troubridge JT (2018) Additions and corrections to the check list of the Noctuoidea (Insecta, Lepidoptera) of North America north of Mexico IV. In: Schmidt BC, Lafontaine JD (Eds) Contributions to the systematics of New World macro-moths VII. ZooKeys 788: 241–252. https://doi.org/10.3897/zookeys.252.28500

Keegan KL, Lafontaine JD, Wahlberg N, Wagner DL (2019) Towards resolving and redefining Amphipyrinae (Lepidoptera, Noctuoidea, Noctuidae): a massively polyphyletic taxon. Systematic Entomology 44(2): 451–464. https://doi.org/10.1111/syen.12336

Keegan KL, Rota J, Zahiri R, Zilli A, Wahlberg N, Schmidt BC, Lafontaine JD, Goldstein PZ, Wagner DL. (2021) Toward a stable global Noctuidae (Lepidoptera) taxonomy. Insect Systematics and Diversity 5(3): 1–24. https://doi.org/10.1093/isd/ixab005

## Patronyms

*Erebia lafontainei* Troubridge & Philip, 1983 [Nymphalidae]

*Eupithecia lafontaineata* Bolte, 1990 [Geometridae]

*Chelis lafontainei* (Ferguson, 1985) [Erebidae]

*Hadena lafontainei* Troubridge & Crabo, 2009 [Noctuidae]

*Euxoa lafontainei* Metzler & Forbes, 2009 [Noctuidae]

*Epidromia lafontainei* Barbut, 2009 [Erebidae]

*Zale lafontainei* Troubridge, 2020 [Erebidae]

*Pelochrista lafontainei* (Wright, 2012) [Tortricidae]

*Lafontaineana* Martinez, 2021 [Noctuidae]

## Additional information

### Use of AI

No use of AI was reported.

### Author ORCIDs

Christian Schmidt https://orcid.org/0000-0003-4160-7629

### Data availability

All of the data that support the findings of this study are available in the main text.

